# Recent Advances in the Management of Patients with Non-Muscle-Invasive Bladder Cancer Using a Multidisciplinary Approach: Practical Recommendations from the Spanish Oncology Genitourinary (SOGUG) Working Group

**DOI:** 10.3390/cancers13194762

**Published:** 2021-09-23

**Authors:** José Rubio-Briones, Ferran Algaba, Enrique Gallardo, José Antonio Marcos-Rodríguez, Miguel Ángel Climent, on behalf of the SOGUG Multidisciplinary Working Group

**Affiliations:** 1 Urology Department, Instituto Valenciano de Oncología & Hospital VITHAS 9 de Octubre, 06009 Valencia, Spain; 2 Pathology Section Fundació Puigvert, Universitat Autònoma de Barcelona, 08025 Barcelona, Spain; falgaba@fundacio-puigvert.es; 3 Oncology Department, Parc Taulí Hospital Universitari, Institut d’Investigació i Innovació Parc Taulí I3PT, Universitat Autònoma de Barcelona, 08208 Sabadell, Spain; egallardo@tauli.cat; 4 Hospital Universitario Virgen Macarena, 41009 Sevilla, Spain; Jose.marcos.sspa@juntadeandalucia.es; 5 Medical Oncology Service, Fundación Instituto Valenciano de Oncología, 46009 Valencia, Spain; macliment@fivo.org

**Keywords:** non-muscle-invasive bladder cancer, microhematuria, transurethral resection, histological variants, molecular subtypes, immunotherapy

## Abstract

**Simple Summary:**

This report presents clinically relevant advances in the management of non-muscle-invasive bladder cancer, which have been the focus of discussion of expert members of the Spanish Oncology Genitourinary (SOGUG) Multidisciplinary Working Group in the framework of the Genitourinary Alliance project (12GU), designed as a space for the integration of novel information in the care of bladder cancer patients. The present study is focused on different aspects regarding the evaluation of hematuria, assessment of second (or repeated) transurethral resection of bladder cancer, histopathological diagnosis and problems with tumor grading, importance of histological variants, shortage of drug supply, and the current role and influence of immunotherapy and biological markers on the oncological outcome of patients. All proposals and recommendations have a multidisciplinary practical approach and are intended to help clinicians in shared decision making for patients with non-muscle-invasive urothelial cancer.

**Abstract:**

On the basis of the discussion of the current state of research on relevant topics of non-muscle-invasive bladder cancer (NMIBC) among a group of experts of the Spanish Oncology Genitourinary (SOGUG) Working Group, recommendations were proposed to overcome the challenges posed by the management of NMIBC in clinical practice. A unified definition of the term ‘microhematuria’ and the profile of the patient at risk are needed. Establishing a ‘hematuria clinic’ would contribute to a centralized and more efficient evaluation of patients with this clinical sign. Second or repeated transurethral resection (re-TUR) needs to be defined, including the time window after the first procedure within which re-TUR should be performed. Complete tumor resection is mandatory when feasible, with specification of the presence or absence of muscle. Budding should be used as a classification system, and stratification of T1 tumors especially in extensive and deep tumors, is advisable. The percentage of the high-grade component should always be reported, and, in multiple tumors, grades should be reported separately. Luminal and basal subtypes can be identified because of possibly different clinical outcomes. Molecular subtypes and immunotherapy are incorporated in the management of muscle-invasive bladder cancer but data on NMIBC are still preliminary.

## 1. Introduction

Bladder cancer is the fourth and the 11th most common cancer in men and women, respectively, representing 6.6% of all cancer types. Non-muscle-invasive bladder cancer (NMIBC) is the most frequent and accounts for about 70–75% of bladder cancers [1]. NMIBC is defined as a neoplasia confined to the mucosa, including Ta (non-invasive papillary carcinoma) and carcinoma in situ (CIS) or lamina propria (T1) based on the American Joint Committee on Cancer (AJCC) staging system [2]. Approximately 60% of patients with NMIBC have Ta tumors, 30% have T1 tumors, and 10% have Tis (CIS) tumors [3]. Despite international comprehensive guidelines for the management of NMIBC [4,5,6,7], emerging diagnostic options, the molecular characterization of urothelial cancer, biomarkers with prognostic capacity, and, in particular, the recurring nature of the disease and long-term survival requiring extensive follow-up and testing procedures pose challenges to clinicians in daily practice and to healthcare systems based on the recognized high costs of the management of these patients.

The Genito-Urinary Alliance project (12GU) was designed as a space for the integration of innovation progress in the management of patients with bladder cancer. To this purpose, expert members of the Spanish Oncology Genitourinary (SOGUG) Multidisciplinary Working Group discussed some controversial and debatable topics of the current knowledge and approach in the care of patients with NMIBC. The aim of the project was to summarize practical recommendations on some particular aspects of NMIBC, including the study of hematuria and referral circuits, difficulties associated with second transurethral resection, clues of pathological diagnosis of tumor grade and subtypes, problems related to stock and supply of bacillus Calmette–Guérin (BCG) for intravesical therapy, and the role of immunotherapy and biological markers. Challenges and recommendations were reached by agreement of all participants to be applicable in clinical practice to facilitate shared decision making for individual NMIBC patients.

## 2. Rapid Diagnosis of NMIBC: Assessment of Hematuria

Painless macroscopic hematuria is the most common presenting symptom of bladder cancer, which occurs in about 85% of patients, followed by irritative symptoms associated with urinary tract infection [1]. In a population-based study of all cases of bladder cancer registered in 2011 by the Spanish Association of Urology at 26 selected hospitals, of a total of 4285 episodes, 2476 (57.8%) were new cases, with a crude incidence rate of 24.4 per 100,000 inhabitants [8]. Clinical symptoms were present in 87.5% of patients at the time of diagnosis, with macroscopic hematuria occurring in 90.8% of cases as the most common manifestation, followed by voiding symptoms in 23.2%, and microscopic hematuria in 17.2%. In 1809 patients with recurrent tumors (incidence rate 17.8 per 100,000 inhabitants), macroscopic hematuria was also the most common symptom among 605 patients (33.5%) with clinical manifestations [8]. In the presence of visible hematuria reported by the patients, a first step is a detailed review of the medical history and thorough physical examination. Gross hematuria is an accepted indication for further investigation because of the risk of significant underlying pathology.

By contrast, the need for investigation of microscopic (nonvisible) hematuria is not well defined, given the high prevalence in the general population (20% in men older than 60 years), dipstick and urine microscopy false positives, different definitions based on age thresholds and patient risk profiles, and multiple underlying etiologies [9]. In a systematic review and meta-analysis to assess the association between microhematuria and the diagnosis of bladder cancer, the pooled detection rates were 3.2% in patients referred for evaluation, 5.3% in studies evaluating men only, and 3.1% in asymptomatic patients [10]. Furthermore, a lower rate of urological referral was shown in patients with microhematuria (36%) as compared to those with visible hematuria (69%) [11]. In a study carried out in the outpatient setting of a public healthcare system in Dallas County of 11,442 patients with urinalysis positive for hematuria, the rate of urological referral was 11.4%, but complete evaluation defined as cystoscopy and ultrasound (US), computed tomography (CT), and magnetic resonance imaging (MRI) (US/CT/MRI) was performed in only 4% of cases [12]. In the logistic regression analysis, age >35 years, male gender, hypertension, and >20 red blood cells (RBCs) per high-power field (HPF) were significantly associated with higher referral to urology [12]. Hematuria is understudied in clinical practice, management is inconsistent, the urological referral rates are low, and there is large room for improvement in the strategies for evaluation of patients with microhematuria.

The first need is to clearly define microhematuria and the situations in which testing is required. Investigation of microhematuria (grade of recommendation C) detects bladder cancer in about 3% of patients (95% confidence interval (CI) 2–5%) and is defined as positive dipstick and a threshold of ≥3 RBC/HPF in the urinary sediment microscopy [10]. A complete anamnesis may reveal potential causes of microhematuria (e.g., benign diseases, nephropathies, anticoagulation, and urine collection conditions). Risk factors should also be taken into account (e.g., smoking, chemicals used in textile, rubber, leather, dye, paint, and print industries, pelvic radiation therapy, and schistosomiasis) so that an underlying disease requiring treatment can be identified or ruled out. Among patients with non-glomerular microhematuria, 80% are idiopathic with no clinical significance, and it is important to differentiate glomerular from non-glomerular microhematuria to establish any algorithm for risk-adapt investigation of asymptomatic microhematuria [13].

**Figure 1:**
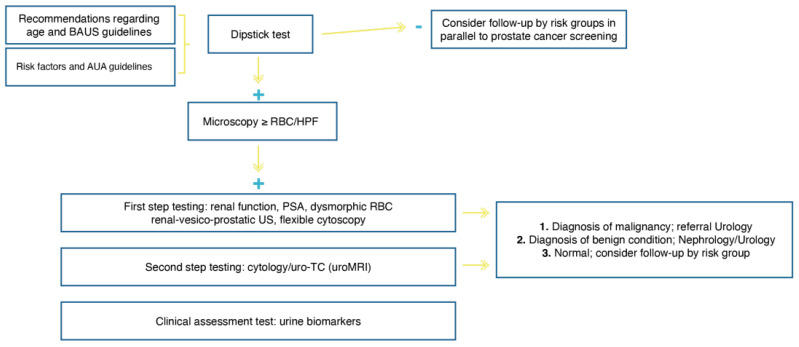
Clinical algorithm for use in a hematuria clinic (BAUS: British Association of Urological Surgeons, AUA: American Urological Association, PSA: prostate-specific antigen, RBC: red blood cells, US: ultrasound, uro-TC: computed tomography urography, uroMRI: magnetic resonance imaging urography).

The second need refers to the establishment of a ‘hematuria clinic’ for the screening and assessment of hematuria according to possibilities of the healthcare system and organizational characteristics of local urology services. An example of a potential clinical algorithm for microhematuria evaluation and urological referral is shown in Figure 1. The regular use of biomarkers to detect bladder cancer, outside clinical trials or registers with research purposes, is not accepted by the European Association of Urology Guideline because their sensitivity is usually higher at the cost of lower specificity compared to urine cytology (level of evidence 3), and the sensitivity and specificity of a urinary marker test largely depend on the clinical context of the patient (level of evidence 3) [4]. An extensive review of the published biomarkers in the context of BT diagnosis is beyond the scope of this paper, although many of them have been proposed in the context of microscopic hematuria, and it is generally accepted that none of the currently available tests can replace cystoscopy.

The third need refers to the establishment of rapid referral circuits based on a diagnostic algorithm for early treatment, differentiation between primary and recurrent bladder tumors, implementation of independent referral circuits for benign conditions, and reference centers for the differentiation of infiltrating tumors and NMIBC. All of these needs should be adapted to the characteristics of each urology service and healthcare system. The following objectives can be pursued in each visit: first visit to the ‘hematuria clinic’ and when a bladder tumor is diagnosed, standard preoperative evaluation, urine culture, and CT urography for tumor staging, which is mandatory in tumors located in the trigone, with multiple lesions or with infiltrating features, and which is recommended before TUR; a second visit performed in less than 15 days including preoperative evaluation, anesthesiology consultation, and preparation for transurethral resection (TUR) of bladder tumors; a third visit within 30 days of TUR.

### Challenges and Recommendations

− To establish a unified definition of the term ‘microhematuria’ and the profile of the patient at risk. On the basis of the evidence, to determine the screening of microhematuria to be used in practice considering the population and the risk factors.
− Creation of ‘hematuria clinics’ in the local healthcare system consisting of a series of specialized centers in which defined diagnostic algorithms and protocols are followed.
− Centralized evaluation in a space in which ultrasound equipment, microscopy, and cystoscopy are available.
− Establishment of a rapid referral circuit adapted to each service.
− Diagnostic and classification algorithm including the referral of patients to the services of urology or nephrology according to the results obtained, with the aim of establishing a rapid referral system adapted to each service.

## 3. Audit Assessment of Repeated or Second TUR

The absence of the detrusor muscle in the first TUR involves a significantly higher risk of residual tumor and downstaging (except for Ta tumors) (level of evidence 2b). Second or repeated TUR (re-TUR) may detect residual tumor and improve downstaging, recurrence-free rate, response to BCG therapy, and prognostic information for the patient (level of evidence 2). There is a strong grade of recommendation to perform re-TUR in the following scenarios: after an incomplete (or doubtful) TUR, if the detrusor muscle is lacking in the specimen of the first TUR (except for Ta tumors), and in all T1 tumors. Moreover, it is advisable to perform re-TUR of the scar between 2 and 6 weeks post TUR and to register histopathological results of re-TUR as a quality pattern of the first TUR.

In a prospective study to evaluate the impact of routine second TUR on the long-term outcome of patients with newly diagnosed pT1 urothelial carcinoma, 191 patients were randomized to second TUR performed within 2–6 weeks of the initial resection (group 1, *n* = 93) or no second TUR (group 2, *n* = 98) [14]. All patients were followed until death or a minimum of 54 months (mean follow-up 66.1 months). Second TUR significantly decreased the recurrence rate (hazard ratio, HR 2.48, *p* = 0.001) and progression rate (HR 3.48, *p* = 0.007) compared to patients who did not undergo a second TUR. In a systematic review of 31 studies including 8409 patients with high-grade Ta and T1 bladder cancer, detrusor muscle was documented at histology of the initial TUR in 30–100% of cases, residual tumor was documented at re-TUR in 20–71% of cases following T1 cancer, and upstaging occurred in 0–32% (T1 to ≥T2) of cases [15]. Despite wide ranges, this review showed that residual tumor is common after TUR for high-risk NMIBC and that re-TUR is of value for diagnosing residual cancer and improving outcomes for cases initially staged as T1. In a large retrospective multicenter cohort study of 2451 patients with T1-high grade/grade 3 bladder cancer, re-TUR in the absence of muscle had a beneficial effect on outcome, including time to recurrence (HR 0.67, *p* = 0.08), progression (HR 0,46, *p* = 0.06), cancer-specific survival (HR 0.31, *p* = 0.07), and overall survival (HR 0.48, *p* = 0.05) [16].

A first need is to distinguish clear definitions for repeated resection (second look) (after multiple bladder cancer leaving residual tumor in the initial TUR), restaging TUR (for clarifying stage in the absence of muscle in the initial resection specimen), and second TUR (re-TUR) after a complete first TUR (with muscle in the initial resection specimen), as well as establishing the timing for re-TUR.

A second need is the implementation of a systematized registry database of the results of initial TUR and re-TUR aimed at collecting data on the rates of muscle detection in the first TUR, second look due to incomplete TUR, re-TUR, and recurrence and progression at 1, 2, and 5 years. Variables to be included in the database for initial TUR and re-TUR include the following: date of surgery; surgeon; number of tumors; location; size of the largest tumor; tumor appearance; primary or recurrent tumor; complete TUR on gross examination; tumor grade; pathologic assessment of the tumor (pT); presence of detrusor muscle; CIS. In relation to systematization and cost–benefit analysis of re-TUR, individual urology services are recommended to perform 6–12 month pilot studies after detailed registration of results and adherence to clinical practice guidelines, adequacy of discharge systems of TUR and control in monographic bladder tumor consultations with referral of results to primary care clinicians, and programming and systematization of hospital admissions for re-TUR when indicated.

### Challenges and Recommendations

− Clear and uniform definitions of re-TUR including the time range within which re-TUR should be performed.
− Improved use of re-TUR in daily practice through systematization of the registry of re-TUR rates corresponding to each service and implementation of a unified protocol for the surgical procedure of re-TUR.
− Establishment of the main healthcare parameters of quality and their periodic audit by multidisciplinary teams.

## 4. Tissue Samples for Histopathology

Careful visual assessment of the whole urothelial lining and excision of all lesions by TUR, with the resection of the underlying bladder wall with the detrusor muscle in order to adequately address the depth of invasion, are emphasized by bladder cancer treatment guidelines [4,5,6] (level of evidence 1). The omission of muscle in TUR specimens of bladder tumors or its mention in the pathological report may negatively impact outcome, particularly in high-grade NMIBC. In this respect, muscle sampling is mainly considered a surrogate marker for the quality of staging in patients with NMIBC.

In an analysis of the clinical records of 1865 patients with bladder cancer from the Los Angeles SEER database (a population-based registry) [17], statistically significant differences (*p* < 0.001) were found in the percentages of patients with muscularis propria present, absent, or not mentioned in Ta tumors (48%, 29.4%, and 22.6%, respectively) and T1 tumors (60.9%, 31.0%, and 8.1%, respectively). Given that urologists cannot make a reliable differentiation between low- and high-grade disease or T1 and Ta disease, adequate muscle sampling at the time of endoscopic resection is a crucial aspect in the management of bladder cancer patients [17]. In another retrospective review of 114 patients with high-grade T1 bladder cancer collected from the Columbia University Urologic Oncology database, the effect of identification of muscularis propria on biopsy specimens before undergoing radical cystectomy was evaluated [18]. The rate of upstaging at the time of cystectomy was 50% if muscularis propria was detected on the initial TUR specimen and 78% if no muscularis propria was identified (*p* = 0.017). In an analysis of 332 patients collected from a bladder cancer database of a tertiary medical center in Tel Aviv, who underwent complete TUR, absence of detrusor muscle in the specimen was associated with higher early disease recurrence in T1 tumors [19].

Taking advantage of the fact that most urothelial carcinomas of the bladder have distinct compositions of the stalk and tumor base (Table 1), an analysis of the pathology records of the University of Washington Medical Center yielded 62 patients with primary lamina propria invasion pT1 tumors, which were divided according to criteria into stalk-invasive only, base focal, and base extensive (20, 13, and 29 patients, respectively) [20]. There were statistically significant differences (*p* < 0.0001) across subtypes in progression-free and event-free survival, with higher risk in the group of patients with base extensive tumors as compared to the two other modalities. This novel approach based on the separation of lamina propria invasion in the substaging of pT1 papillary tumors may reflect differences in anatomical, compositional, and biological aspects of stalk as compared with base stromal invasion [20].

**Figure 2:**
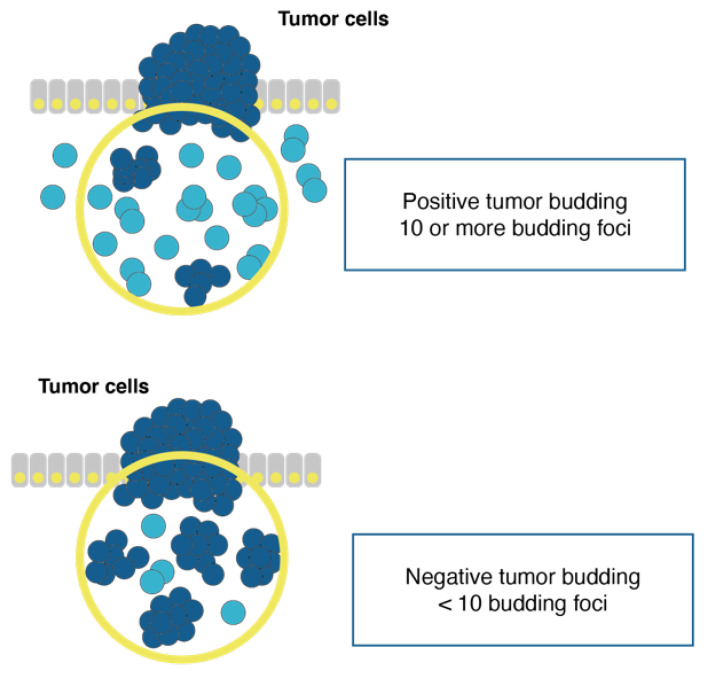
Schema of tumor budding. The circle represents an area of magnification, showing ≥10 (positive) or <10 (negative) budding foci of isolated single cancer cells or a cluster composed by a few cancer cells (modified from Fukumoto et al. [21]).

The concept of tumor budding is illustrated in Figure 2 and consists of a single cancer cell found isolated or a few cancer cells forming a cluster scattered in the stroma. Tumor budding was used to assess the prognostic impact of budding on outcome in 121 patients with T1 NMIBC treated at Keio University Hospital in Tokyo [21]. Tumor budding, defined as the presence of ≤10 foci, was a significant risk factor for 5 year progression-free survival (53.8% in tumor budding-positive patients vs. 88.4% in tumor budding-negative patients, *p* = 0.001). On the other hand, in a review of 616 patients with stage T1G2 treated with TUR and followed for a mean of 4.2 years, variables associated with progression to muscle-invasion disease were recurrence at 3 months (relative risk (RR) 4.0, 95% CI 1.2–13.3, *p* = 0.02), high-grade disease or CIS at first recurrence (RR 2.8, 95% CI 1.3–5.8, *p* = 0.005), and CIS associated with primary tumor (RR 1.8, 95% CI 1.1–2.9, *p* = 0.009); furthermore, high-grade disease or CIS at first recurrence and CIS associated with primary tumor were predictors of recurrence at 6 months [22].

### Challenges and Recommendations

− Complete tumor resection should be routinely performed when feasible.
− The presence or absence of muscle should always be specified, as well as the extension of infiltration; in case of doubt, it is necessary to use different microscopic levels of the block for a definite diagnosis.
− Systematized substaging assessment method.
− Stratification of T1 bladder tumors, especially in extensive and deep tumors.
− Use of budding as a classification system and a possible predictive indicator of stage progression in T1 tumors.
− Quality parameters should be established and periodically reviewed by multidisciplinary teams.

## 5. Problems with Histological Grading

**Figure 3:**
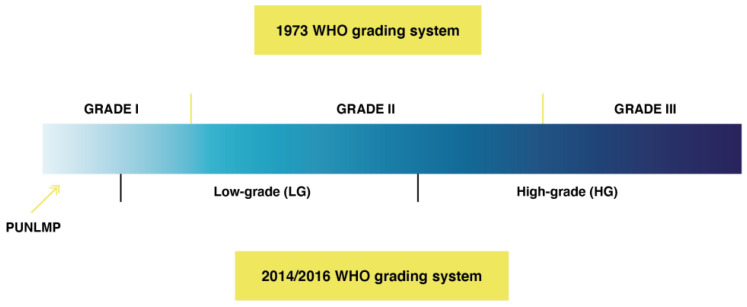
WHO grading systems for non-muscle-invasive bladder cancer. (PUNLMP: papillary urothelial neoplasms of low malignant potential).

Grading has particular clinical relevance in NMIBC, and different clinical guidelines [3,4,5,6] may recommend pathology reporting using the 1973 and 2004/2016 WHO grading systems, with G1–G3 or low-grade (LG)/high-grade (HG) and the category of papillary urothelial neoplasms of low malignant potential (PUNLMP) (level of evidence 2a) (Figure 3).

In relation to reporting the presence of a minor (<5%) HG component in an otherwise LG urothelial cancer, a review of 31 mixed-grade urothelial carcinomas revealed that mixed-grade carcinomas with less than 5% HG component had a significantly better stage progression-free survival than pure HG urothelial tumors [23]. Furthermore, a reasonable approach would be to perform grading based on the highest grade and, if this HG component is present in more of 10%, it is advisable to communicate the HG percentage in the pathology report [24].

In a study of 3311 patients with primary Ta bladder tumors from 17 hospitals in Europe and Canada, the prognostic values of Ta low-grade (Ta-LG) and Ta high-grade (Ta-HG) tumors were analyzed [25]. Time to progression was similar for PUNLMP and Ta-LG with significant differences as compared with Ta-HG (*p* < 0.01); progression at 5 years of follow-up was 2.6% (95% CI 0–5.7) in the papillary urothelial neoplasm of low malignant potential (PUN-LMP), 2.1% (95% CI 1.3–1.9) in the Ta-LG, and 7.5% (95% CI 5.5–9.5) in the Ta-HG groups, respectively. Considering these findings, the consideration of PUNLMP as a differential grade category in Ta tumors does not appear to be justified because the prognosis of patients with PUNLMP and Ta-LG is similar. Moreover, in another study of 5145 patients with primary Ta/T1 NMIBC tumors collected from the same 17 hospitals in Europe and Canada, it was found that the group of G2 subdivided into the LG and HG categories had different prognostic implications, with progression rates at 5 years of 7.7% and 18.8%%, respectively (*p* < 0.01) [26]. In a recent consensus of the International Society of Urological Pathology, the splitting of the WHO 2004 high-grade category into WHO 1973 grade 2 and 3 subsets is recommended [27].

### Challenges and Recommendations

− Use of strict criteria of architectural cytological order/disorder and nuclear size.
− In mixed-grade urothelial carcinomas, the highest grade (>10%) should be indicated, as well as the percentage of the different grades if the highest grade is <10%.
− In high-grade tumors, the G3 component should be indicated.
− In multiple tumors, grades should be reported separately.
− In the case of PUNLMP, clinical management should be the same as if Ta-LG had been reported.
− Grading systems should have prognostic value in Ta and T1 tumors.

## 6. Importance of the Different Subtypes

During the last decade, there has been an increasing interest in the assessment of histological variants in bladder cancer, such as plasmacytoid, nested, large nested, microcystic, tubular, micropapillary, lipoid cell, sarcomatoid, microcystic, clear cell, lymphoepithelioma-like, and others [28]. However, the role of histological variants in cancer-specific mortality and the possibility that different types of differentiation have distinct outcomes remain unclear, given the small populations of cases with histological variants in case series studies (level of evidence 3). A systematic review and meta-analysis that included 13 trials with a total of 9533 participants with bladder cancer showed that histological variants did not predict a poor prognosis, and squamous/non-squamous differentiation did not influence survival [29]. However, since most variants are infiltrating subtypes, it is difficult to assess whether any variant has more aggressive behaviors per se. Being aware that there are controversial data regarding the prognostic value of histological variants, our recommendation is to consider them as a potential sign of aggressive behavior.

In relation to molecular characterization, different studies have shown the existence of two distinct molecular subtypes of bladder cancer referred to as luminal and basal. According to hierarchical clustering of basal and luminal biomarkers using whole-transcriptome expression data from independent cohorts of 937 bladder cancer samples, two signature markers, one luminal (GATA3) and the other basal (KRT5/6), were sufficient to classify bladder cancer into the categories of basal and luminal with 90% accuracy [30]. Survival analyses confirmed that invasive basal tumors were consistently more aggressive when compared to luminal tumors and were associated with significantly shorter survival than the luminal counterparts [30].

**Figure 4:**
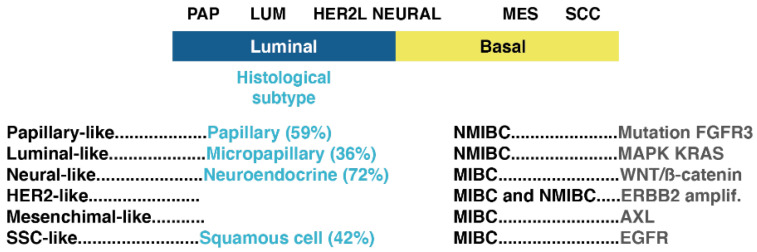
Molecular subtypes and the corresponding histological subtypes (frequency in parenthesis). PAP: papillary; LUM: luminal; MES: mesenchymal; SCC: squamous-cell carcinoma-like; NMIBC: non-muscle-invasive bladder cancer; MIBC: muscle-invasive bladder cancer (modified from Tan et al. [31]).

In an analysis of the bladder carcinoma dataset with 2411 tumors encompassing muscle-invasive bladder cancer (MIBC) and NMIBC, the subtypes of which were assigned by gene expression reproduced on the Cancer Genome Atlas, UROMOL, and IMvigor210, six molecular subtypes with different molecular features and overall survival were identified [31] (Figure 4). These subtypes included NEURAL (neural-like), LUM (luminal-like), PAP (papillary-like), HER2L (HER2-like), MES (mesenchymal-like), and SCC (squamous-cell carcinoma-like), which corresponded to histological subtypes of papillary for papillary-like in 59% of cases, micropapillary for luminal-like in 36% of cases, neuroendocrine for neural-like in 72% of cases, and squamous cell for SCC-like in 42% of cases. The subtype neural-like is prevalent in MIBC and characterized by high WNT/β catenin signaling, the HER2-like is distributed in MIBC and NMIBC with higher ERBB2 amplification, the papillary-like is an NMIBC subtype with a high frequency of FGFR3 mutations, the luminal-like is predominantly in NMIBC with higher MAPK signaling and KRAS mutations, and the mesenchymal-like and SCC-like are predominant in MIBC, with high AXL signaling in mesenchymal-like and elevated PD1 and CTLA4 signaling in SSC-like [31].

Molecular subtype may also be heterogeneous, a fact that complicates translation of molecular subtyping into patient care. In a study of 309 cystectomy cases for bladder cancer, molecular subtyping was performed on the histological variants, specifically, squamous, glandular, micropapillary, nested, plasmacytoid, and sarcomatoid, as well as conventional urothelial carcinomas co-occurring with these variants [32]. It was found that 93% of tumors were classified as urothelial-like, genomically unstable, or basal squamous, and, in patients with more than one tumor histology, 39% showed molecular heterogeneity, which was greater for the basal-squamous subtype [32].

Lastly, therapeutic indications based on histological classification of bladder cancer are tentatively summarized in Table 2.

### Challenges and Recommendations

− The pathology report should include the tumor subtype classification as different treatment approaches are needed according to specific subtypes.
− The molecular subtypes have been mostly described in MIBC, and the evidence in NMIBC is limited.
− Luminal and basal molecular subtypes are well recognized. The basal subtype has oncological therapeutic connotations.

## 7. Drug Supply Shortage

In relation to drug supply shortage described in this section, we refer to the situation in Spain as an example of what could happen in healthcare systems from other countries. There is a drug supply shortage when drug units available in the pharmaceutical channel are inferior to the local or national consumption needs. In this situation, healthcare authorities of Spanish autonomous communities and marketing authorization holders (as an obligation) must communicate this circumstance to the Spanish Agency of Medicines and Medical Devices (AEMPS). The total number of notifications to the AEMPS related to drug supply problems increased from 20 in 2008 to 1332 in 2018, the main reasons being difficulties in the supply of active principles, increasing demand of drugs, manufacturing and capacity problems, and quality-related issues [33]. In order to guarantee the 2019–2022 medicine supply, the AEMPS has developed a plan with the assistance of healthcare professionals, patient associations, laboratories, and distributors [34]. Measures to be taken are aimed at preventing shortage of essential products, early detection and therapeutical substitution, penalty revision, and improvements in reporting and monitoring systems.

In relation to mitomycin-C, the main problems were associated with delays in the packaging fabrication plant and increasing demand. In the case of BCG, Inibsa Hospital interrupted the supply due to quality problems, with the demand unable to be supplied by other laboratories. There are special situations considered by the AEMPS in which commercial alternatives to these drugs may be requested, such as Misintu 20 mg (Turkey), mitomycin Medac 40 mg (Norway/Denmark), CG culture SSI 30 mg Deco Pharma, BCG-Medac Gebro Pharma, or OncoTICE, directly requested by MSD on a monthly basis.

### Challenges and Recommendations

− Collaboration with the regulatory agencies (AEMPS).
− Establishment of an active and early warning system in order to have sufficient time for managing the implementation of alternative solutions.
− Pharmacotherapeutic and clinical management with unified protocols.
− Selection of preferential indicators, coordination of multidisciplinary teams for the management of information, and promotion of the role of the “active” and “trainer/expert” patient.

## 8. Role of Immunotherapy and Biological Markers

Immunotherapy in MIBC has focused on checkpoint inhibitors, including programmed cell death protein-1 (PD-1) and programmed death ligand-1 (PD-L1), which offer an effective second-line alternative for patients previously treated with platinum-based regimens [35,36,37,38,39,40,41,42], a first-line treatment for cisplatin-ineligible patients [35,36], or maintenance treatment in patients who showed no progression with first-line chemotherapy [43]. Results of different studies showed a higher overall response in PD-L1-positive than in PD-L1-negative patients (Table 3), although differences in survival were not found. An association has been shown between higher PD-L1 expression and higher objective response rate (ORR), but no biomarker has been established for selecting bladder cancer patients for immune-oncology therapy.

The IMvigor130, a phase 3 clinical trial carried out in untreated patients with metastatic urothelial carcinoma, supports the use of atezolizumab combined with cisplatin-based chemotherapy as first-line treatment for metastatic bladder cancer, with the use of atezolizumab monotherapy in selected patients [43]. In the PD-L1 IC2/3 subgroup, the survival curves favored atezolizumab; however, in the PD-L1 IC0/1 subgroup, the survival curves favored platinum-based chemotherapy earlier, with atezolizumab ultimately appearing to cross the curve. In the JAVELIN Bladder 100 trial of advanced urothelial carcinoma [44], maintenance avelumab also significantly prolonged overall survival in the PD-L1-positive population. Overall survival at 1 year was 79.1% in the avelumab group and 60.4% in controls (best supportive care alone); the median progression-free survival was also more favorable in the avelumab arm.

In order to determine a reliable biomarker to predict which patients could benefit from immune checkpoint inhibitors (PD-1/PD-L1) using a large set of genomic data from The Cancer Genome Atlas (TCGA), a classification into four groups of tumors and their microenvironments was established. This classification was based on expression of PD-L1 and recruitment of tumor-infiltrating lymphocytes (TIL), as evaluated by the expression of CD8A [45]. In the PD-L1^+^/CD8A^+^ (TMIT I) group, which was characterized by high PD-L1 expression, potentially favorable predictive factors of response to treatment with PD-1/PD-L1 drugs included high MSI (microsatellite instability), high mutational burden/neoantigen, the presence of an oncogenic virus, and PD-L1 amplification [45].

In a phase 2 study that examined the efficacy of two cycles of atezolizumab before cystectomy in patients with MIBC, the presence of immune infiltrate with CD8^+^ cells and PD-L1 positivity were associated with a higher probability of survival [46]. Another study showed that identification of a T-cell-inflamed tumor microenvironment was possible with the presence of concordance with CD8^+^ T cell infiltration, and that T-cell-inflamed tumors were associated with upregulation of genes encoding immune checkpoint proteins PD-L1, FOXP3, IDO, TIM3, and LAG3, suggesting potential sensitivity to immune checkpoint blockade [47]. Moreover, in a study of tumors from patients with metastatic bladder carcinoma treated with atezolizumab (an anti-PD-L1 drug), it was found that high tumor mutation burden or neoantigen and, to a lesser extent, the CD8^+^ T-effector cell phenotype were associated with treatment response [48].

Table 4 shows the main characteristics of the six molecular subtypes of MIBC: luminal papillary, luminal non-specified, luminal unstable, stroma-rich, basal/squamous, and neuroendocrine-like, which accounted for 24%, 8%, 15%, 15%, 35%, and 3% of cases, respectively [49].

Each molecular subtype is associated with overexpression or decreased expression following mutation of different biomarkers. In general, molecular alterations described in subtypes with higher genomic instability (luminal unstable, basal, and neuroendocrine profiles) are related to a better response to immunotherapy.

Data on biomarker assessments. including expression of individual genes and gene sets, as well s subtyping according to TCGA, have been reported in various clinical trials. In the study of atezolizumab as first-line therapy in cisplatin-ineligible patients with locally advanced metastatic urothelial carcinoma, there was a significantly higher tumor mutation burden in patients who showed a response than in non-responders, with this finding being consistent across PD-L1 subgroups and TCGA subtypes [35]. Overall survival also showed an association with mutation burden, whereby patients in quartile 4 (the highest mutation burden) as compared to patients in quartiles 1 to 3 had significantly longer survival times [35].

In a large group of patients with metastatic bladder cancer who received atezolizumab, a signature of transforming growth factor β (TGFβ) signaling in fibroblasts was significantly associated with non-response and reduced overall survival [48]. In the ABACUS trial [45], tGE8 expression (a transcriptional signature of eight genes) was enriched in responders vs. those with progressive disease. In the CheckMate 025 trial of nivolumab in metastatic urothelial cancer, the analysis of efficacy by baseline biomarkers showed higher response rates in basal 1 (cluster 3) (complete response 8.7%, partial response 21.7%) and luminal 2 (cluster 2) (partial response 25.4%) than in the other two subtypes (luminal 1, partial response 16.6%; basal 2, partial response 15.1%) [50]. Transcription profiling of 368 tumor samples from the IMvigor 210 trials of atezolizumab in platinum-refractory or cisplatin-ineligible patients with urothelial carcinoma identified a neuronal subtype in a small group of patients with a very high survival probability, which may be secondary to low levels of TGFβ expression and high mutation/neoantigen burden [51].

### 8.1. BGG-Unresponsive NMIBT

This is a group of major interest because of higher progression rates, broadly characterized by any high-grade disease occurring during or after BCG therapy, while differentiating NMIBT that may not respond at all (BCG refractory) from NMIBT relapsing after initial response (BCG relapsed). There is a paucity of data of treatment with PD-L1 inhibitors in high-risk NMIBC patients. In the single-arm phase 2 KEYNOTE-057 study [52], the effect of pembrolizumab was evaluated in patients with high-risk BCG-unresponsive NMIBC who declined to undergo or were ineligible for cystectomy and were treated with pembrolizumab 200 mg every 3 weeks for 24 months or until recurrence, progression, or unacceptable toxicity. Results were updated (median follow-up 36.4, range 32.0–40.7) and, at 3 months, in the group of 96 patients with carcinoma in situ unresponsive to BCG, with or without papillary tumors, 41% (*n* = 39) showed a complete response. In 13 patients (13%), grade 3 or 4 adverse events related to the treatment occurred, with hyponatremia and arthralgia being the most frequent (3% and 2% of cases, respectively). In 8% of patients, treatment-related adverse events were serious, but no deaths were recorded [52].

The single-arm phase 2 SWOG S1605 trial communicated the effect of 1 year treatment with atezolizumab (1200 mg/3 weeks) in 74 patients with carcinoma in situ unresponsive to BCG therapy. Furthermore, a complete response was observed in 31 (42%; 95% CI 31–54) patients at 3 months and in 20 (27%; 95% CI 17–39) patients at 6 months [53]. Grade 3 or 4 treatment-related adverse events appeared in 15 (16%) patients, and one patient died due to sepsis with respiratory failure after myasthenia gravis.

Pembrolizumab added to BCG therapy for patients with high-risk NMIBC persistent or recurrent disease after induction therapy with BCG is being evaluated in an ongoing phase III trial (KEYNOTE-676) [54]. The aim of the trial is to assess the efficacy and safety of this combined treatment.

Other ongoing phase III clinical trials with the checkpoint inhibitors durvalumab (NCT 03528694) and atezolizumab (NCT 03799835) combined with BCG instillation in BCG-naïve patients are aimed at evaluating the efficacy and safety of these agents in the NMIBC setting.

### 8.2. Challenges and Recommendations

− Incorporation of immunotherapy in the management of NMIBC.
− The precise indications and opportunities of immunotherapy in the management of high-risk NMIBC should be defined, differentiating unresponsive patients to BCG therapy from BCG-naïve patients.
− Definition of predictive factors/profiles of benefit from immunotherapy, as well as the value of PD-L1.
− Validation of factors and molecular subtypes in infiltrating tumors.
− Multidisciplinary collaboration to develop NMIBC registries with clinical data and biological sample collections (biobanks) for improving molecular aspects of NMIBC.

## 9. Conclusions

It remains clear that optimal management of NMIBC is challenging, and a multidisciplinary approach is needed in both clinical and molecular research. There is room for improvement in diagnosis and urological strategies, but the present recommendations have to be adapted to and optimized in each healthcare system. Pathologists play a key role in the characterization and risk profiling definition; a pathologist specialized in these areas is highly recommended. Healthcare providers, authorities, and clinicians have to be on the same page during drug shortages. Lastly, molecular subtyping and immunotherapy have yet to demonstrate their role and cost-effectiveness in both BCG-naïve and BCG-unresponsive HG patients, and we all have to support translational and clinical research toward this aim.

## Figures and Tables

**Table 1:** Papillary urothelial carcinomas (pT1) with invasion of lamina propria [20].

Categories	Histological Features
Stalk invasion only	− Single cells with rounding outpouching of different size − Irregular tumor nests confined to the papillary stalk. − Loose edematous connective stroma. − Frequent paradoxical differentiation and retraction artefacts.
Base invasion focal	− Single focus of microinvasion within 1 high-power field (HPF) at 20× corresponding to 1 mm, characterized by the following: − Condensed and eosinophilic base connective stroma. − Noticeable *muscularis mucosae* fascicles or prominent vasculature (9t is advisable to distinguish between above *muscularis mucosae* or into *muscularis mucosae*). − Prominent vasculature. − Increased inflammatory infiltrate. − Sometimes marked desmoplastic response.
Base invasion extensive	− Few microinvasive foci more than 1 mm apart. − Present in multiple tissue fragments. − Invasion of an area that would not fit within 20 HPF.

**Table 2:** Histological classification of bladder cancer and therapeutic approach.

Tumor Classification	Management Recommendation
Low-grade	Surveillance
High-grade	BCG/immunotherapy *
Ta	Surveillance
T1	According to stratification
CIS	BCG/immunotherapy *
T2–4	Cystectomy/chemotherapy/immunotherapy
Luminal-like papillary	Fibroblast growth factor receptor (FGFR) inhibitors *
Luminal-like unstable	Immunotherapy
Basal/squamous	Chemotherapy/immunotherapy

* High degree of uncertainty.

**Table 3:** Immunotherapies for first-line and second-line metastatic urothelial carcinoma: PD-L1 expression and response.

Immunotherapy Agent [Reference]	No. of Patients	Definition PD-L1^+^ Expression	PD-L1^+^ %	Objective Response Rate (ORR), %		
				Overall	PDL-1^+^	PDL-1^−^
First-line treatment						
Atezolizumab [35]	119	IC2/3	27	23	28	21
Pembrolizumab [36]	370	CPS ≥ 10	30	29	51	23
Second-line treatment						
Atezolizumab [37]	310	IC2/3	32	15	26	8
Avelumab [38]	249	≥5% TC	?	17.4	25.4	13.2
Durvalumab [39]	191	≥25% TC or IC	66	17.8	27.6	5.1
Nivolumab [40]	270	≥1% TC	46	19.6	23.8	16.1
Pembrolizumab [41]	270	CPS ≥ 10%	27.4	21.1	21.6	NR
Atezolizumab [42]	467	IC2/3	25	13	23	NR
Maintenance in patients with no progression after first-line chemotherapy						
Avelumab [43]	350	NR	54	71.3 *	79.1 *	NR

IC: tumor-infiltrating immune cells; CPS: combined positive score; TC: tumor cells; NR: not reported. * Overall survival at 1 year (after randomization).

**Table 4:** ACGT consensus molecular subtypes of MIBC tumors [49].

Characteristics	Urothelial/Luminal				Basal	Neuroendocrine
	Luminal Papillary	Luminal Non-Specified	Luminal Unstable	Stroma-Rich	Basal/ Squamous	Neuroendocrine-Like
Oncogenic mechanisms	FGFR3 + PPARG + CDKN2A −	PPARG +	PPARG + E2F3 +, ERBB2 + Genomic instability, cell cycle +		EGFR +	TP53 −, RB1 − Cell cycle +
Mutations	*FGFR3* (40%) *KDM6A* (38%)	*ELF3* (35%)	*TP53* (76%) *ERCC2* (22%), TMB +, APOBEC +		*TP53* (61%) *RBI* (25%)	*TP53* (94%) *RB1* (39%)
Stromal infiltrate		Fibroblasts		Smooth muscle Fibroblasts Myofibroblasts	Fibroblasts Myofibroblasts	
Immune infiltrate				B cells	CD8 T cells NK cells	
Histology	Papillary morphology (59%)	Micropapillary variant (36%)			Squamous differentiation (42%)	Neuroendocrine differentiation (72%)
Clinical	T2 stage +	Older patients + (80+)			Women + T3/T4 stage +	
Overall survival, years, median	4	1.2	2.9	3.8	1.2	1

## Data Availability

Data supporting recommendations and collected by consensus of the expert members of the Spanish Oncology Genitourinary (SOGUG) Working Group are available upon request to the authors.
